# Assignment of serotype to *Salmonella enterica* isolates obtained from poultry and their environment in southern Brazil

**DOI:** 10.1111/lam.12110

**Published:** 2013-06-24

**Authors:** M Pulido-Landínez, R Sánchez-Ingunza, J Guard, V Pinheiro do Nascimento

**Affiliations:** 1 Avian Pathology Laboratory, College of Veterinary Medicine, National University of ColombiaBogotá, Colombia; 2 Avian Diagnostic and Research Center, College of Veterinary Medicine, Federal University of Rio Grande do Sul (UFRGS)Porto Alegre, Brazil; 3 United States Department of Agriculture, Agricultural Research ServiceAthens, GA, USA

**Keywords:** epidemiology, food safety, genotype, poultry, *Salmonella enterica*, serotype

## Abstract

To assess diversity of *Salmonella enterica* serotypes present in poultry and their environment from southern Brazil, the Kauffmann–White–Le Minor (KWL) scheme was used to serotype a total of 155 isolates. Isolates were then re-examined with nested PCR and sequencing of the *dkgB*-linked intergenic sequence ribotyping (ISR) region that assesses single nucleotide polymorphisms occurring around a 5S ribosomal gene. Serotypes identified were Heidelberg (40·6%), Enteritidis (34·2%), Hadar (8·4%), Typhimurium (3·9%), Gallinarum (3·2%), Agona (1·3%), Cerro (1·3%), Livingstone (1·3%), Infantis (0·6%), Isangi (0·6%), Mbandaka (0·6%), Montevideo (0·6%) and Senftenberg (0·6%). Three unique ISRs were detected from four strains. Day old chicks yielded only *S. *Enteritidis, whereas *S*. Heidelberg was most often associated with poultry carcasses. Overall agreement between KWL and ISR was 85·2%, with disagreement possibly due to the ability of ISR to detect mixtures of serotypes in culture. Overall, ISR provided more information than did KWL about the ecology of *Salm. enterica* on-farm. The O-antigen group D *Salm*. *enterica* serovars such as Pullorum, Gallinarum and Enteritidis appear susceptible to overgrowth by other serotypes.

**Significance and Impact of the Study:**

Single nucleotide polymorphisms found in a group of poultry-associated *Salmonella* isolates from southern Brazil provided evidence of mixtures of serovar group D serotypes on-farm and in single samples from birds. This finding suggests that co-infection and interserotype competition of *Salmonella enterica* in poultry could impact the incidence of disease in animals or humans. In addition, unique serotypes were identified on-farm that escaped characterization by antibody typing. Application of cost-efficient and highly discriminatory genomic methods for assigning serotype may alter concepts about the epidemiology of *Salm. enterica* on-farm and in foods.

## Introduction

Brazil currently ranks as the major exporter of chicken meat in the world with markets in more than 150 countries. *Salmonella enterica* (*Salm. enterica*) is one of the most important bacterial pathogens that can cause foodborne illness (Schroeder *et al.* 2006). It is often transmitted to people by eggs and other poultry products (Hogue *et al.* 1997). The Brazilian poultry industry is committed to the control of *Salmonella* and its impact on the health of birds and the safety of poultry products. The industry follows the guidelines listed in the National Poultry Health Plan that includes monitoring of *Salm. enterica* on farms and in poultry products. (www.agricultura.gov.br/arq_editor/file/Aniamal/Manual). Efforts to control *Salm. enterica* in poultry are complicated by differences in the predominant serotype, strain heterogeneity, prevalence, biosecurity practices, extent of regulatory control and the nature, size and logistic complexity of this industry (Mead *et al.* 2010). The increasingly strong demand for safe poultry products requires application of diagnostic tools that enable rapid and reliable identification of pathogenic micro-organisms.

An obstacle to maintaining the safety of poultry products is the difficulty in accessing reliable diagnostic tools to characterize *Salm. enterica* as part of a continuously operational monitoring programme. Serotyping is an important epidemiological tool in the characterization of *Salm. enterica*, because it helps determine the prevalence and emergence of pathogenic serotypes in different regions. The Kauffmann–White–Le Minor (KWL) scheme is the historical method for determining serotype (Grimont and Weil 2007; Hendriksen *et al.* 2009). It is antibody-based and uses a combination of agglutination reactions to determine serotype. More than 2600 serotypes have been described on the basis of the antigenic structure of the cell surface lipopolysaccharide (O antigen) and flagellar proteins (H antigen; Grimont and Weil 2007). The KWL scheme is prone to subjective interpretations arising from combinatorial complexity, mixtures of serotypes in culture, and the absence of cell surface epitopes that are targeted by antisera (Guard *et al.* 2012). A lack of information about which serotypes are circulating on-farm impedes measures for effective treatment and control because different serotypes have different niches (Dorneles *et al.* 2010). Molecular methods for subtyping strains have potential for assigning serotype in reference to the historical database developed from the KWL scheme, because serotype is a class of strain variation and thus results from variation in the genome of *Salm. enterica* (Murase *et al.* 1995; McQuiston *et al.* 2008; Wattiau *et al.* 2011; Achtman *et al.* 2012; Fabre *et al.* 2012). Pulsed-field gel electrophoresis (PFGE) is considered a pivotal DNA-based method that complements the KWL scheme in regard to detecting strain variation occurring within serotype, and it has been used successfully to identify origins of outbreaks. Other DNA-based methods can be used to subtype and may also provide serotype information, and each one has its advantages and disadvantages (Wattiau *et al.* 2011). Common disadvantages of most DNA-based methods are the need to purchase proprietary equipment, reagents and software for analysis (Tankouo-Sandjong *et al.* 2008; Wise *et al.* 2009; McQuiston *et al.* 2011). Overall, most molecular techniques are restricted to centralized public health laboratories with infrastructure that supports objectives to conduct research and epidemiological studies.

Intergenic sequence ribotyping (ISR) is a rapid strain typing method that lowers barriers to implementation because costs for equipment and software are modest (Guard *et al.* 2012). It is sequence-based and has been shown to distinguish between serotypes at the level of the single nucleotide polymorphism. Cultures with more than one serotype can be detected when forward and reverse sequences do not align. The attributes of low cost, simplicity of application and the ability to access reference sequences suggested that ISR was suitable for application to widespread use for environmental studies. The objective of the present study was to determine if sequence-based ISR analysis of 155 isolates of *Salm. enterica* isolated from poultry and their environment from southern Brazil would yield additional information in comparison with those obtained using KWL serotyping. Results suggest that the ecology of *Salm. enterica* has aspects that have not yet been considered in determining risks to the safety of the food supply.

## Results and discussion

Intergenic sequence ribotyping performed with specific primers assigned a *Salm. enterica* serotype to 151 of 155 samples collected from poultry in southern Brazil (Tables1 and 2). The KWL serotype initially reported was confirmed for 85·2% (132/155) of the samples. A total of 15 serotypes were identified, which included *S*. Enteritidis*, S*. Heidelberg*, S*. Hadar*, S*. Typhimurium*, S*. Gallinarum*, S*. Agona*, S*. Senftenberg*, S*. Livingstone*, S*. Cerro*, S*. Infantis*, S*. Mbdanka*, S*. Montevideo*, S*. Isangi and three different unique sequences (UN0041, UN0042 and UN0043). Of the 155 samples, 23 (14·8%) had an ISR that did not agree with initial KWL serotyping (Table2). Of the samples reported as *S*. Heidelberg, two were identified by ISR as *S*. Enteritidis and two as unique sequence UN0042. *S*. Hadar had one observed difference, which was *S*. Heidelberg. In the four samples reported as *S*. Agona, two sequences were assigned *S*. Isangi and UN0043. For 8 *S*. Typhimurium identified by KWL, two were assigned *S*. Heidelberg by ISR (Table2).

**Table 1:** Primers used for nested PCR intergenic sequence ribotyping (ISR)

Primer Name	Orientation	Primer sequence (5′ to 3′)	Reference	Amplicon size (bp)
ISR-F1	Forward	GCCAATGGCACTGCCCGGTA	Guard *et al*. (2012)	1464
ISR-R1	Reverse	TACCGTGCGCTTTCGCCCAG	Guard *et al*. (2012)	
nISR-F14	Forward	TGCCCCGAGATGAGTTCTCCC	This study	1187^a^
nISR-R14	Reverse	CACCCGGAGATGGCCAGTGGAT	This study	
ISRs1_F8	Forward	AGGCCGGGTGTGTAAGCGCA	This study	–
ISRs2_R42	Reverse	CGGAACGGACGGGACTCGA	This study	

a Size of amplicon may vary as much as 250 bp between serotypes.

**Table 2:** Variation between reported serotype and serotype as detected by ISR analysis

Reported serotype	Number of samples	Number confirmed as reported by ISR	Number of other ISRs detected	Other ISR serotypes detected (number per ISR is in parentheses)
Heidelberg	62	58 (93·5%)	4 (6·5%)	Enteritidis_499 bp (2) UN0042_365 bp (2)
Enteritidis	54	48 (88·9%)	6 (11·1%)	Heidelberg_498 bp (1), Mbandaka_11813C_499 bp (1), Livingstone_11841_ 361 bp (1) Infantis_9381_500 bp (1) Cerro_5767N_502 bp (2)
Hadar	14	13 (92·9%)	1 (7·1%)	Heidelberg_498 bp (1)
Gallinarum	10	5 (50·0%)	5 (50·0%)	Heidelberg_498 bp (1) Enteritidis_499 bp (1) Montevideo_6690_362 bp (1) Senftenberg_362 (1) Livingstone_11841_361 (1)
Typhimurium	8	6 (75·0%)	2 (25·0%)	Heidelberg_498 bp (2)
Agona	4	2 (50·0%)	2 (50·0%)	Isangi_13416_258 bp (1) UN0043_399 bp (1)
Pullorum	3	0	3 (100·0%)	Enteritidis_499 bp (2) UN0041_361 bp (1)
Total	155	132 (85·2%)	23 (14·8%)	–

ISR, intergenic sequence ribotyping.

Three avian-associated serovar group D *Salmonella* serovars, namely Enteritidis, Gallinarum and Pullorum, had several disagreements between the KWL serotype and ISR (Table2). Of the 67 samples, 14 (20·9%) disagreed with KWL serotyping. Of the other 88 isolates that were not group D, nine had disagreement between KWL serotype and ISR (10·2%) (Table2). *S*. Pullorum and *S*. Gallinarum are avian pathogens with a fastidious growth habit that can require longer incubation times. Of the 13 samples in this collection, eight (61·5%) were putatively mixed as detected by an ISR indicating the presence of a different serotype (Table2). These results suggest that the group D Salmonellae may be prone to overgrowth by minority serotypes present within culture during storage. Alternatively, the group D Salmonellae may have a propensity to associate with other serotypes, and thus, overgrowth is a related outcome. Further research using competition experiments would be required to understand why so many isolates of the group D Salmonellae yielded a secondary serotype. In three cases, *S*. Enteritidis was isolated after either *S*. Gallinarum or *S*. Pullorum had been isolated (B138, B143 and B145). For B137, *S*. Heidelberg was identified by ISR, but *S*. Gallinarum was found by KWL serotype. This result supports that mixtures of serotypes in culture were common.

*Salmonella* Agona was isolated four times from commercial layer organic farms in 2010, and these results were confirmed by PFGE (Tables2 and 3). In this study, DNA samples identified as B133 and B134 were again classified as *S*. Agona by ISR (498 bp), whereas B154 and B155 were not. It is possible that PFGE analysis was not sensitive enough to detect differences between the two *S*. Agona strains and the other two isolates. However, sequence alignment indicated that disagreement of both B154 and B155 ISRs with the one from *S*. Agona was substantial, and no alignment was possible at 90% similarity. These results suggest that two isolates of *S*. Agona were overgrown with serotype *S*. Isangi in one case and UN0043 in the other.

**Table 3:** *Salmonella enterica* serotypes from southern Brazil as assigned by ISR in reference to the Kauffmann–White–Le Minor scheme

Reported serotype	Source of isolation of *Salmonella enterica*									
	Poultry carcass	Drag swab	1-day-old Broiler	Cloacal swab	Organic farm	Feed	Liver	Pips	Unknown	Total
Heidelberg	42	4	–	11	–	1	1	–	3	62
Enteritidis	16	22	13	–	–	2	–	1	–	54
Hadar	14	–	–	–	–		–	–	–	14
Gallinarum	–	–	–	–	1	1	–	–	8	10
Typhimurium	–	1	–	–	1	–	–	–	6	8
Agona*	–	–	–	–	4	–	–	–	–	4
Pullorum	–	–	–	–	–	–	–	–	3	3
Total	72	27	13	11	6	4	1	1	20	155

* Strains also identified by pulsed-field gel electrophoresis (PFGE) (Perdoncini, 2011). www.lume.ufrgs.br/bitstream/handle/10183/36857/000819160.pdf.

Four sources of isolates included over ten samples, and these were poultry carcass (73), drag swab (27), cloacal swab (11) and 1-day-old chick (13) (Table3). The 1-day-old chick samples yielded only *S*. Enteritidis, and there were no mixtures. Cloacal swabs had only *S*. Heidelberg by KWL serotyping, but one sample (B94) may have been mixed with UN0042. Serotypes from drag swabs were 81·5% *S*. Enteritidis by KWL serotyping with one sample putatively mixed with *S*. Mbandaka (B15). The remaining samples were *S*. Heidelberg with one sample putatively mixed with *S*. Typhimurium (B146). Poultry carcasses had the most complicated *Salmonella* flora. According to the KWL scheme, 43 of 73 samples (58·9%) were *S*. Heidelberg, 16 (21·9%) were *S*. Enteritidis and 14 (19·2%) were *S*. Hadar. Of the three serotypes initially recovered from poultry carcasses, 5 of 16 (31·3%) *S*. Enteritidis cultures may have been mixed. In contrast, 7·1 and 4·7% of samples initially found to have *S*. Hadar and *S*. Heidelberg were putatively mixed. These results suggest that *S*. Enteritidis was frequently overgrown by other *Salmonella* serotypes when stored. An alternative explanation is that *S*. Enteritidis had a propensity to associate with other serotypes.

Intergenic sequence ribotyping analysis revealed substantially new information about the collection of 155 isolates from southern Brazil that could impact the assessment of risk for emergent foodborne illness in humans or disease in poultry on-farm. The most frequent contributor to disagreement between KLW serotyping and ISR in this study, as it was previously, was the presence of mixed serotypes in culture (Guard *et al.* 2012). For example, detection of multiple serotypes present when single colonies are passaged strongly suggests that the human pathogen *S*. Enteritidis circulates with the avian pathogens *S*. Gallinarum and *S*. Pullorum on-farm. This finding contradicts the concept that closely related *Salm. enterica* serotypes are mutually exclusive (Baumler *et al.* 2000). It is conceivable that a flock could be infected with two serotypes at one time, and co-infection could affect the percentage of contaminated eggs that enter the market or the health of a flock. Further research is needed to address the impact of co-infection of chickens with serovar group D avian-associated *Salmonella*, especially as it impacts internal egg contamination, public health and disease in chickens.

DNA microarray was used for samples that had unique ISRs because references were not yet available in sequence databases (Guard *et al.* 2012). Serovars *S*. Mbandaka, *S*. Montevideo, *S*. Livingstone, *S*. Infantis and *S*. Cerro were thus assigned to ISRs that are now part of a reference database maintained at the United States Department of Agriculture. Serotypes, *S*. Mbandaka and *S*. Infantis, are considered emergent *Salmonella* serotypes in Brazil with low frequencies of isolation from poultry products (4·8 and 7·6%, respectively) (http://www.anvisa.gov.br/alimentos/relatorios/relatorioprebaf.pdf). The other three serotypes have not yet been reported as prevalent serotypes in Brazil. These results indicate that ISR can be used to find serotypes that may not necessarily have a whole genome reference, but that are represented within DNA microarray hybridization databases.

Serotypes may differ in how they are impacted by storage and by how likely they associate with other serotypes. For example, storage of *S*. Enteritidis at −80°C caused a shift in phenotypes as assayed by composition of the outer membrane, but storage of *S*. Typhimurium did not (Parker *et al.* 2001). Another complication of evaluating stored cultures is that a change could occur in the relative prevalence of mixtures of serotypes over time (Borsoi *et al.,* 2009, Oliveira *et al.,* 2002). Access to a less expensive, efficient and highly discriminatory technique for serotyping that can find mixtures should enable appropriate decisions to be made to improve the safety and security of the food supply.

## Materials and methods

### Bacterial strains

A total of 155 isolates of *Salm. enterica* subsp. *enterica* obtained from different poultry sources were evaluated in this study (Table2). Strains were provided by the Avian Diagnostic and Research Center at the Federal University of Rio Grande do Sul (CDPA – UFRGS for acronym in Portuguese; Table2). Bacteria were isolated from poultry facilities located in the state of Rio Grande do Sul in southern Brazil between 1995 and 2010. Serotyping, using the KWL scheme, was done by the National Reference Institute Oswaldo Cruz/FIOCRUZ. Strains were conserved at −80°C in 20% glycerol until analysis by ISR.

### Preparation of *Salmonella* DNA

*Salmonella* strains were revived from freezer stocks in 5 ml of brain–heart infusion (BHI) broth by transferring one 10-μl loop of frozen cells in the media and statically incubating the suspension for 24 h at 37°C. Cultures were streaked on XLD and XLT4agar and incubated for 24 h at 37°C to obtain separated colonies. One colony was transferred to BHI broth and incubated overnight at 37°C. Cell culture was diluted to match a McFarland Turbidity Standard 0·5, and 150 μl of the cell suspension was applied to one circle on Whatman FTA ® classic cards (GE Healthcare Bio-Sciences Corp., Piscataway, NJ, USA). FTA cards were subsequently shipped with an APHIS permit for importation and transportation of controlled materials for analysis at the United States Department of Agriculture, Agricultural Research Service, Egg Safety and Quality Research Unit; (USDA-ARS-ESQRU) in Athens, GA, USA (Pulido-Landinez *et al.* 2012).

### DNA isolation

Whatman FTA® cards were processed following manufacturer instructions with some refinements (Pulido-Landinez *et al.* 2012). Briefly, discs were excised from the cards using a 3·00 mm Harris Uni-Core device and transferred in to sterile 1·5-ml-Eppendorf tubes before adding 200 μl of FTA purification reagent. To avoid cross-contamination between samples, the collecting device was cleaned by cutting two discs from a noninoculated FTA card. Tubes were vortexed for 5 s and incubated at 25°C for 5 min at 450 rpm using an Eppendorf Thermomixer (Eppendorf, Hamburg, Germany). The purification reagent was discarded and replaced with fresh reagent to repeat this step one more time. Discs were then washed twice with 200 μl of TE buffer under the similar conditions indicated for the purification reagent and discarding the buffer in between washes. TE buffer was completely removed before continuing with PCR analysis. Safety testing was conducted on each DNA sample adsorbed in the FTA cards in order to determine if embedded reagents killed bacteria. One disc was placed in BHI broth and incubated with shaking for 24 h at 37°C. If turbidity was observed, culture was streaked on brilliant green (BG) agar (Acumedia, Neogen Corporation, Lansing, MI, USA) to test for the presence of *Salmonella*.

### PCR analysis and sequencing of DNA

Nested polymerase chain reaction (N-PCR) method was used to obtain PCR products for analysis of sequence. N-PCR and sequencing primers were designed using the Primer-Blast designing tool from the National Center for Biotechnology Information (NCBI; Tables1 and 2). Purified DNA in FTA discs was amplified using primers ISR-F1 and ISR-R1 in the first PCR and primers nISR-F14 and nISR-R14 in the second PCR. Briefly, FTA discs were transferred to 0·2-ml-PCR tubes containing 2× Gene Amp Fast PCR Master Mix (Applied Biosystems, Foster City, CA, USA) and 200 nmol of primers ISR-F1 and ISR-R1 in a volume of 30 μl. One microlitre of the PCR product from this reaction was transferred in to 29 μl of Master Mix solution containing primers nISR-F14 and nISR-R14 in the same aforementioned primer concentrations. Amplification was performed on a Veriti 96-well Fast Thermal cycler (Applied Biosystems) under the following cycling conditions: initial denaturation at 95°C for 10 s followed by 35 cycles of 94°C for 0 s, 64°C for 40 s and 72°C for 10 s with a final extension at 72°C for 10 s. DNA products from the second PCR were resolved in 2% ethidium bromide agarose gels (Invitrogen, Carlsbad, CA, USA). Then, the amplified DNA in those samples containing the expected nucleotide size was purified using a PureLink® PCR purification kit (Invitrogen). DNA concentrations, ranging from 15 to 50 ng/uL, were measured in a NanoDrop ND-1000, Spectrophotometer (NanoDrop, Wilmington, DE, USA), and purified PCR products were submitted to Retrogen, Inc. (San Diego, CA, USA) for DNA sequencing in an ABI Prism 3730 DNA analyzer (Life Technologies, Grand Island, NY, USA) using primers ISRs1_F8 and ISRs2_R42 (Table1).

### Construction of intergenic sequence ribotyping sequences and data analysis

The ISR sequence starts from the nucleotide located immediately after the 23S rRNA gene and the nucleotide located immediately before the tRNA-*asp*U gene in the *Salmonella* genome region linked to the *dkg*B gene (Morales *et al.* 2006). Finding no ambiguous nucleotides within at least 300 base pairs (bp) of remaining sequence after ends were trimmed assessed the quality of sequence data. In addition, substantial overlap of forward and reverse sequences consisting of at least one flanking intergenic sequence and some of the 5S rRNA gene should be observed. Forward and reverse sequencing data corresponding to each individual sample were aligned using SeqMan Pro Lasergene v8.0 (DNASTAR, Madison, WI, USA), and ISR sequences were assigned serotype. ISR sequences were assigned serotype using SeqMan Pro after a 100% similarity match with the ISR sequence database for *Salmonella* serotyping maintained at the USDA-ARS-ESQRU was observed (Guard *et al.* 2012).

### Analysis of intergenic sequence ribotyping sequences with no available sequence database

The DNA hybridization microarray (DNAhyb) *Salmonella* serotyping method is AOAC RI certified and marketed as Check&Trace (Checkpoints, Wageningen, the Netherlands) was used to resolve *Salmonella* serotype identity of the unique ISR sequences detected in this study (Guard *et al.* 2012). DNAhyb was modified to analyse DNA extracted from FTA discs (Pulido-Landinez *et al.* 2012), because international restrictions on shipment of live pathogens encourages shipment of DNA only. *Salmonella* DNA was prepared as suggested by E. Mundt (personal communication). Briefly, three FTA discs were placed into a 1·5-ml-Eppendorf tube containing 0·5% SDS and 0·5 mg ml^−1^ Proteinase K in 180 μl distiled water. Tubes were briefly vortexed and incubated at 55°C for 60 min at 450 rpm in an Eppendorf Thermomixer. DNA was extracted from the liquid fraction using a PureLink™ Genomic DNA kit from Invitrogen and following the protocol for Gram-negative bacteria cell lysates after proteinase K digestion. DNA concentrations were measured in a NanoDrop ND-1000 Spectrophotometer (NanoDrop) and 10 μl of extracted DNA were added into the PCR reaction tubes provided within the kit. DNAhyb was otherwise followed as indicated by the manufacturer.
